# Treatment of Acute Psychosis with Second-Generation Antipsychotics in a Patient with Left Temporal Lobe Lesion

**DOI:** 10.1155/2018/9839252

**Published:** 2018-02-05

**Authors:** Richard Shehane, Steve Miller, Luke Suber, Miranda Chakos

**Affiliations:** Department of Psychiatry, Naval Medical Center Portsmouth, Portsmouth, VA, USA

## Abstract

We present a case of rapid onset severe psychosis followed by suicide attempt in a United States Navy sailor. Investigation revealed a left temporal lobe brain mass suspicious for low-grade glioma. After hospitalization and medical management with olanzapine and lurasidone the patient's psychosis improved. The purpose of this paper is to add to the existing case reports that suggest a relationship between temporal lobe lesions and psychiatric illness, specifically psychosis. In addition, this case adds insight into the effectiveness of medical therapy for brain tumor patients that are not immediate candidates for neurosurgical intervention.

## 1. Introduction

Brain and central nervous system (both malignant and nonmalignant) tumors are relatively common with an average annual age-adjusted incidence of 28.57 per a population of 100,000 in the United States. For comparison, prostate and breast cancer are the most common cancers among those aged 20+ years in the United States, with average annual age-adjusted incidence rates of 192.83 per population of 100,000 (males only) and 172.01 per population of 100,000 (females only), respectively. In addition, brain and central nervous system tumors are the most common cancers among those aged 0–19 years, with an average annual age-adjusted incidence rate of 5.57 per population of 100,000 [1].

Brain tumors often present with focal neurologic deficits due to mass effect. However, rare cases may present solely with psychiatric symptoms including depression, apathy, mania, psychosis, personality changes, and eating disorders. Madhusoodanan et al. (2015) recently reviewed literature from 1956 to 2014 regarding 172 brain tumors where the primary manifestation was psychiatric. Of these 172 cases, 30 cases presented with psychosis with three cases specifically confined to the left temporal lobe. Many of these cases were managed with surgery. Meanwhile, the remainders were managed medically with therapy tailored towards their specific symptoms [2].

## 2. Case

A 21-year-old male United States Navy sailor with no past medical history presented to the emergency department after attempting to commit suicide by jumping off the flight deck of an aircraft carrier into the ocean. Weeks prior to his suicide attempt, the patient noticed difficulty completing mundane tasks such as brushing his teeth and bathing and increased forgetfulness, such as forgetting the location of his parked car. Days prior to the incident he started to become extremely restless, unable to sleep, and developed auditory hallucinations, including musical hallucinations and voices commanding him to kill himself.

**Figure 1:**
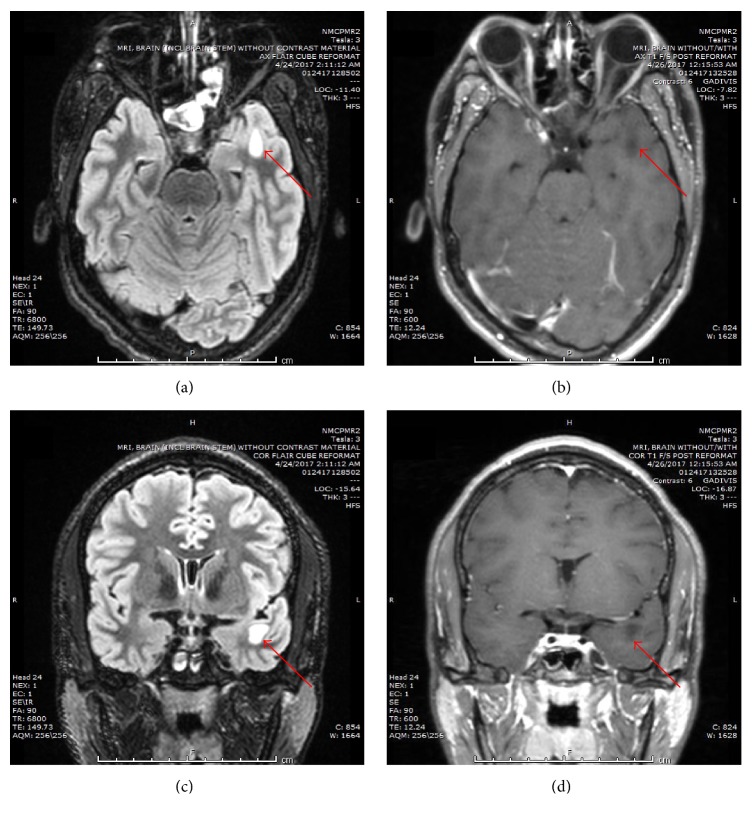
Brain MRI demonstrating left temporal lobe lesion. (a, c) Axial and coronal T2 weighted images. (b, d) Axial and coronal T1 weighted images. Red arrow points to lesion.

Primary investigation revealed unremarkable laboratory studies and drug screens. Magnetic resonance imaging (MRI) of the brain showed an area of T1 hypointensity and T2 hyperintensity involving predominantly the white matter of the upper aspect of the left temporal lobe anterior tip. This area showed no post-intravenous contrast enhancement and measured approximately 2.5 × 1 cm, sagittal dimensions × 1.2 cm, transverse (Figure 1). The patient was evaluated by the neurosurgical team which opted to delay surgery in favor of repeat MRI at a later date to track progression of the tumor.

The patient's first mental status examination demonstrated a spontaneously alert black male who appeared his stated age. He was pleasant and cooperative with the interview and maintained appropriate eye contact. He appeared restless, despite having been given 2 mg of lorazepam approximately one hour prior to evaluation. His speech was spontaneous with slow rate, normal rhythm, low tone, and low volume. He described his mood as “feeling better”; affect was tired and seemed congruent with his stated mood. His thought process was disorganized, with loosening of association. At the time of interview he denied suicidal/homicidal ideations and visual hallucinations, although he still reported hearing the same song continuously repeating in his mind. No systematized delusions were noted. On mini-mental state examination, the patient had difficulty with the attention and calculation focused task of serial sevens [3]. He also had difficulty with working memory tasks such as naming the months of the year backwards. The patient was able to do abstractions of common proverbs. Insight was poor with poor recall of recent suicide attempt, judgment was impaired, and impulse control was poor. He scored 21/30 on an initial Montreal Cognitive Assessment (MOCA) with deficits for hands of clock, attention, memory, and orientation [4].

Over the course of his hospitalization the patient was treated with olanzapine which was titrated up to 15 mg per os (PO) daily. Initially he continued to exhibit thought blocking, concentration problems, and word finding difficulty, as well as difficulty carrying out simple tasks (e.g., “taking a shower”) and disorganized thinking. However, by hospital day 14, the patient was beginning to improve with a repeat MOCA scoring 28/30, and neuropsychological cognitive testing demonstrating a cognitive profile not consistent with a significant cognitive impairment.

By hospital day 30 the patient was switched to lurasidone due to a 7 kg weight gain. He continued to improve during his hospitalization without recurrence of auditory hallucinations or suicidal ideations. In this case the neurosurgical team opted for monitoring with serial MRIs. The patient responded well to medical therapy. He was monitored on the inpatient psychiatric ward for a total of 61 days and eventually discharged on lurasidone 40 mg PO bid.

## 3. Discussion

There are numerous studies that propose a link between structural abnormalities of the temporal lobes and psychosis [2, 5, 6]. In the case of a temporal lobe tumor, surgical resection can be beneficial in that it provides tissue for histologic and genetic classification, as well as relief of mass effect symptoms. Some studies have noted improved survival in patients with maximal resection of their low-grade glioma; however, there may be bias in these studies as these patients were compared to patients in which maximal resection was not a viable option [7, 8].

In addition, amelioration of psychiatric symptoms after surgical resection of a temporal lobe tumor has not been well studied and in some cases temporal lobe resections have been shown to be the cause of psychotic symptoms [9, 10]. Therefore, immediate surgery may not always be the best option and generally is only absolutely indicated for patients with severe neurological deficits or a large mass.

The other option is watchful waiting with serial imaging, avoiding intervention unless intractable seizures, progressive neurologic impairment, or transformation to a high-grade glioma develops. However, the pitfall of this strategy is that surgical resection may no longer be feasible if the lesion grows too aggressively between monitoring periods [11]. Thus, a definite area of controversy still remains in the management of these patients. In cases such as ours with a small tumor and no focal neurological deficits it was decided that the patient was best served initially with antipsychotic medications alone.

## 4. Conclusion

The connection between psychiatric illness and brain tumors can be easily overlooked. It is always worthwhile to pursue cranial imaging in a patient presenting with their first episode of psychosis. When lesions are found on imaging surgical intervention is not always the immediate answer, especially in a young patient with a suspected low-grade glioma. This case bolsters previous case reports that suggest a relationship between the left temporal lobe and psychosis and demonstrates the effectiveness of second-generation antipsychotics in the treatment of brain tumor induced psychosis. Further studies are warranted to establish the best surgical approach in patients with small, low-grade gliomas and symptoms controlled with medication.
